# The Minimalist Program and the Origin of Language: A View From Paleoanthropology

**DOI:** 10.3389/fpsyg.2019.00677

**Published:** 2019-04-02

**Authors:** Ian Tattersall

**Affiliations:** Division of Anthropology, American Museum of Natural History, New York, NY, United States

**Keywords:** evolution, paleoanthropology, origins, exaptation, language and thought

## Abstract

In arguing that articulate language is underpinned by an algorithmically simple neural operation, the Minimalist Program (MP) retrodicts that language emerged in a short-term event. Because spoken language leaves no physical traces, its ancient use must be inferred from archeological proxies. These strongly suggest that modern symbolic human behavior patterns – and, by extension, cognition – emerged both abruptly and late in time (subsequent to the appearance of *Homo sapiens* as an anatomical entity some 200 thousand years kyr ago). Because the evidence is compelling that language is an integral component of modern symbolic thought, the archeological evidence clearly supports the basic tenet of the MP. But the associated proposition, that language was externalized in an independent event that followed its initial appearance as a conduit to internal thought, is much more debatable.

## Introduction

Spoken language is famously ephemeral. In the absence of preserved writing systems language leaves no direct trace in any material record, so that its putative employment by hominids at virtually any point in human prehistory has to be inferred from indirect proxy evidence. One might hope that the fossil record of early humans would be helpful here; but in the event, this turns out to be a problematic line of inquiry. There is obviously a relationship of some kind between the morphology of the upper vocal tract (roofed by the skull base) and the physical ability to produce the sounds used in the articulate speech through which we express language; but the nature of that relationship remains highly controversial (Lieberman, 2012; Fitch et al., 2016). What is more, while we may safely conclude that any fossil hominid displaying the distinctively modern human retracted-face splanchnocranial anatomy had possessed the potential for speech production, it is far from evident that the potential for speech necessarily implies a concomitant possession of language. For these reasons, putative proxies for speech and language use are more usefully furnished not by fossils, but by the archeological record, our only first-order archive of ancient human behaviors. Frequently, though, archeological evidence offers us no more than shadowy or indirect traces of the full complexity of past human behaviors. And in consequence it is particularly important that any conclusions we draw from it about when and how our human precursors acquired language should align appropriately with the broader contexts in which we might reasonably expect this unique human property to have arisen. Those key contexts include both what we know of evolutionary pattern in general, and what we know of the intrinsic properties of language itself.

In an ideal world, contextual considerations of both kinds would have acted as constraining influences on our perceptions of when and how language evolved, and on the kinds of proxy evidence for it that might be considered satisfactory. But in practice they seem to have done little to limit the variety of language proxies that different observers have been willing to accept; and the resulting lack of agreement has quickly polarized, leaving very little middle ground between the possible extremes. On the one hand, there are those who believe that language is so complex and multifaceted that it can only have emerged over a very long period, under the guiding hand of natural selection. This would have occurred, by implication almost inevitably, as complex societies and sophisticated vocal communication gradually co-evolved. On the other side are those who think that language merely involved a tweak to prior systems of vocal communication and mental information processing, and that the transition from non-linguistic to linguistic was rapidly accomplished, in a single bound.

In the first camp are those aligned with Stephen Pinker and Paul Bloom, who roundly declared a quarter-century ago that “every detail of grammatical competence … must have conferred a reproductive advantage on its speakers … and there must be enough time and genomic space separating our species from non-linguistic primate ancestors” (Pinker and Bloom, 1990: 745). In the other camp are those who broadly agree with Derek Bickerton that “true language, via the emergence of syntax, was a catastrophic event, occurring within the first few generations of *Homo sapiens*” (Bickerton, 1995: 69). Of late, Lieberman (2013, 2015) has been a particularly energetic advocate of the former view, whereas Bolhuis et al. (2014, 2015) have equally vigorously defended the latter one. In terms of the absolute time-scale involved in language acquisition, some authorities (e.g., Uomini and Meyer, 2013) would equate the neural processes underpinning language with those underlying Acheulean stone-working techniques, thereby extending the rudiments of language back to almost two million years ago. In contrast, those accepting the spirit of Bickerton’s declaration (including this author: see Tattersall, 2012) would look for the abrupt acquisition of full-blown language at some time under 200 thousand years (kyr) ago, when the first modern humans appeared.

The Minimalist Program (MP: Chomsky, 1993, 1995) sees language as underpinned by an algorithmically simple interface between sensorimotor and conceptual-intentional systems that were co-opted from pre-existing functions or potentials. The MP thus has clear and immediate relevance to the long-running dispute over language origins, being very comfortably compatible with – indeed, predicting – the notion that language as we recognize it today originated suddenly, at some definable point in the human past. By itself, the MP does not predict the timing of this event; but its emphasis on algorithmic simplicity coexists uneasily, at best, with the idea that language is simply too rich and too complex to have been achieved quickly and that it must therefore have evolved gradually and incrementally over the eons. Accordingly, in the absence of any clearly articulated alternative, independent demonstration that language was acquired in a short-term event may be taken as support for the MP’s tenets.

## The Material Record

The merits of the two major opposing viewpoints continue to be debated among linguists and others on a variety of intrinsic grounds; but from a paleoanthropological perspective the choice between the longer and shorter timescales, or between the gradualist and punctuationist models of origin, will most obviously be resolved by extrinsic archeological evidence. Most proponents of gradualism rely on evidence for behavioral “complexity,” in one form or another, as proxy for linguistic status. Complex behaviors such as grinding ochre, the production of lithic utensils (at varying stages of sophistication), and the non-functional modification of objects, have all been taken at one time or another as behaviors sufficiently complex to imply that their practitioners had language. The problem here, though, is that there are evidently many different ways in which to be an intelligent hominid with complex behaviors, not all of them necessarily involving linguistic skills. What is more, most of the purely functional behaviors that we associate with the Paleolithic simply do not appear to have mapped directly on to the unique modern cognitive mode with which we can firmly associate language (Tattersall, 2012). In seeking indicators of modern cognitive status, we thus need to look beyond strictly technological and economic activities in the material record and instead to focus on evidence for the kinds of behaviors that are uniquely governed by the modern human style of information processing to which we can reasonably correlate language.

The most fundamental distinguishing property of modern human cognition lies in its “symbolic” nature, the term deriving from the fact that – just as in language – the individual’s external environment and internal mental states are deconstructed by the thought process into a vocabulary of discrete symbols that can be recombined, according to rules, to make statements about the world not only as it is directly perceived by the senses, but as it *might* be. The human symbolic capacity, in other words, imposes an arbitrary discreteness on what is otherwise a perceptually continuous world, allowing the entities thereby distinguished to be both mentally manipulated and conceptually extended. And because the elements of symbolic thought map closely onto the vocabularies of words that we use as linguistic building-blocks, it is highly probable that we are justified in using anything we can legitimately regard as a routine material expression of symbolic mental operations as a proxy for the hominid possession of language. This view fits very well with the perspective of the clinical and theoretical linguist Wolfram Hinzen, who has recently argued very persuasively that language and thought are not “two independent domains of inquiry,” (Hinzen, 2012: 640), and has more specifically espoused the notion that thought is inherently grammatical. In consequence, only where we have evidence for symbolic thought can we confidently conclude we have evidence for language.

One category of objects that might legitimately be regarded as proxies for modern cognition, and hence for language, is the overtly symbolic: representational images, for example, or plaques bearing engraved signs. But since spoken language is a community possession, we also need evidence that objects which might individually be seen in this light were actually integrated into a larger symbolic tradition: evidence that is best furnished by the existence of multiple examples within the same archeological context. The production of symbolic objects is, moreover, only one offshoot of the modern cognitive capacity. Combined with high technological skills, the routine ability to imagine that the world might be other than it currently is should reasonably be expected to express itself in a distinctive pattern of innovation in the archeological record. After all, cognitively modern human beings have radically altered the face of the planet in a geological eyeblink, so that at the start of the process of acquisition a detectable inflection in the material record would reasonably be expected.

From the very beginnings of the archeological record, technological innovation was invariably followed by an extended period of stasis lasting from hundreds of thousands of years to over a million (Tattersall, 1998). But at around 100 kyr ago, a hundred millennia after the appearance in Africa of anatomically modern *Homo sapiens* (McDougall et al., 2005), a pattern of additive change was clearly beginning to assert itself within the cultural period known to African archeologists as the Middle Stone Age (MSA). Ochre was being mixed into pigments (Henshilwood et al., 2011); and marine shells, pierced for stringing into bodily ornaments and often bearing traces of pigment, occur at several sites (e.g., Bouzouggar et al., 2007; d’Errico et al., 2009). By around 80 kyr we find multiple geometrically engraved plaques at Blombos Cave in South Africa (Henshilwood et al., 2002, 2009), a site which has also yielded evidence for sophisticated multi-stage material-hardening technology (Mourre et al., 2010) – one of very few Paleolithic technologies that almost certainly mandated symbolic planning abilities. The nearby Pinnacle Point caves also have evidence for fire-hardening by 72 kyr or even earlier (Brown et al., 2009), and they also furnish indications of complex forward planning in the exploitation of marine resources (Marean, 2014). Hard on the heels of these developments, around 70 kyr ago, modern humans expanded beyond the confines of Africa and rapidly took over the Old World, driving all resident hominid competition to extinction by about 40 kyr ago (see Tattersall, 2012), a point at which we also see the initiation of a long and extraordinary tradition of cave art and other symbolic activities, not only in Europe but also in Sulawesi (Aubert et al., 2014) and Borneo (Aubert et al., 2018). In a remarkably short time, change replaced stasis as the default mode in the archeological record: rather than adapting old tools to new uses when environmental exigency demanded, humans were inventing new ones. A revolution in the relationship of humans to the world around them had been accomplished, in a mere few tens of thousands of years.

As the late Pleistocene began, *Homo sapiens* was far from the only species of *Homo* around; and the best-known of its congeners were undoubtedly clever and resourceful. So perhaps it is unsurprising that one of them would have occasionally produced an object of a kind that a symbolic hominid might also have made: a mollusk shell some 500 kyr-old with zig-zag-engraving, putatively the work of *Homo erectus* (Joordens et al., 2014); eagle talons notched for stringing by early *Homo neanderthalensis* (Radovčić et al., 2015); a deep hash engraving probably made by late Neanderthals (Rodríguez-Vidal et al., 2014); some paint on Spanish cave walls that arguably predated Cro-Magnon arrival (contrast Hoffmann et al., 2018 with Slimak et al., 2018). But it is important to appreciate that all such expressions are floating points, and that there is no wider archeological context suggesting that symbolic reasoning of the modern human kind was an established characteristic of any Neanderthal society. The Neanderthals left behind them a record suggesting that they were without doubt intelligent and resourceful. But there are evidently many ways to be a clever hominid; and, despite a few straws in the wind (e.g., Rodríguez-Vidal et al., 2014; Radovčić et al., 2015), this record does not suggest that the symbolic manipulation of information that is best equated with language was a routine component of the Neanderthal cognitive repertoire (Tattersall, 2012). Indeed, the overwhelming message of the extensive Neanderthal archeological record is that these sophisticated and large-brained hominids related to their environments, and to each other, in a very different way from the Cro-Magnons who replaced them. Smart they and their non-modern contemporaries unquestionably were; but there is evidently more than one way in which to be a highly intelligent hominid, and only in the case of *Homo sapiens* do we have clear evidence of a cognitive revolution in the late Pleistocene. The most convincing evidence for this is furnished by the undisputable fact that, once anatomically modern humans began to show evidence for a radically new behavioral pattern, they rapidly left their natal continent and took over the entire Old World, substantially replacing resident hominids wherever they went – *Homo erectus* in Asia, *Homo neanderthalensis* in Europe – and everywhere substituted a cultural pattern of restless and continual change for the ancestral pattern of technological uniformity interspersed with rare innovations.

So how did this extraordinary transition occur, involving as it did a qualitative leap and not simply an incremental cognitive improvement of the kind that probably accompanied earlier advances? Well, because there can be little doubt that the switch from intuitive to symbolic cognition was extremely rapid, the simplest proximate explanation appears to be that the radical developmental reorganization that gave rise some 200 kyr ago to the highly distinctive *Homo sapiens* skeletal anatomy had also, exaptively, produced what Boeckx and Benitez-Burraco (2014) have termed a “language-ready brain,” one possessing the internal connections required to make the complex associations that are involved both in language and symbolic thought. This new brain evidently continued to function in the ancestral intuitive manner for some 100 kyr, until its enhanced associative potential was recruited for symbolic thought by what was necessarily a behavioral stimulus – much as ancestral birds only tardily recruited their feathers for flight. Most probably the stimulus concerned involved a spontaneous attribution of meaning to specific vocal sounds, initiating a mental feedback process between sound and meaning. This created a cascade of associations, governed by rules, that in turn eventuated in language and structured thought (Tattersall, 2012, 2017). But whatever the exact mechanism may have been, we know it initiated a rapid transition; and the simultaneous origin of language and symbolic thought suggested here not only conveniently obviates any chicken-and-egg arguments, but also makes it much easier to understand the intimate relationship between the two that Hinzen (2012) noted. Interestingly, the new symbolic cognitive algorithm seems to have proven more energetically frugal than its intuitive predecessor in which cognitive complexity almost certainly scaled with brain size, since the human lineage subsequently witnessed a significant reduction in neural volume (Tattersall, 2018).

Placing the acquisition of language within the tenure of *Homo sapiens* as an anatomically distinctive entity has another very significant advantage. For years, researchers from Laitman et al. (1979) to Fitch et al. (2016) have lustily argued over the relevance of hyoid and cranial base morphologies to the ability to produce the sounds necessary for articulate speech: a debate that is rendered entirely superfluous if, as suggested here, the modern vocal tract anatomy was in fact already in place before it was co-opted for the production of speech. The same, of course, would also apply to arguments over the relevance of other putatively speech-associated structures such as Broca’s cap (see Falk, 2014). The neural and cranial morphologies necessary for speech production were there first – as, indeed, they had to be.

## The Minimalist Program

The MP describes a research strategy based on the proposition that, as a “finite computation system yielding an infinity of expressions” (Berwick and Chomsky, 2016: 1), language depends on a simpler mental algorithm than many had expected. In doing so, it pares universal grammar down to the minimum essentials necessary to meet the conceptual and phonological requirements of the human beings who, uniquely, use it (Boeckx, 2006). The MP thus has implications for the evolutionary roots of human language, predicting that it had a single origin in time as the result of a simple algorithmic flip, rather than emerging gradually over the eons. This is entirely consonant with the archeological evidence discussed above, which strongly suggests that modern language-based cognition emerged not only suddenly, but also late in time, subsequent to the emergence of anatomically recognizable *Homo sapiens.*

In their most recent articulation of the MP, Berwick and Chomsky (2016) acknowledge this. But they go on to argue not only that Merge, the underpinning operation that combines objects into new syntactic units, was acquired by hominids in Africa around 80 kyr ago, but that the resulting internalized “language of thought” was “*at some later stage* … connected to the sensorimotor system” (2016: 87; italics added) to produce spoken language as a means of communication. As the product of an avowedly minimalist approach, this elaborate two-stage process – internalization first, externalization later – seems oddly unparsimonious. And it also robs us of any explanation for why “the minor biological change that provided the operation Merge” should have become fixed, or in what context the new system might have become co-opted for externalized expression. It hardly seems enough to remark simply that this latter complex task “could have been solved in many ways and at different times” (Berwick and Chomsky, 2016: 87). In contrast, we know that it is entirely possible for modern human beings with language-ready brains to extemporaneously begin attaching meaning to symbol (Senghas et al., 2005), resulting in the emergence of a structured language readily capable of rapid subsequent refinement. In the case just cited the currency of the new language was visual signs; but vocal symbols are clearly even more effective in this role since they are not limited to line-of-sight. In conjunction with the necessary possession of an exaptively language-ready brain, it is this associative aptitude of our species that makes the spontaneous invention of spoken language, at some point around 100 kyr ago, by far the most credible putative driver of symbolic thought and modern cognition.

## Author Contributions

IT wrote the article.

## Conflict of Interest Statement

The author declares that the research was conducted in the absence of any commercial or financial relationships that could be construed as a potential conflict of interest.
